# RAA-CRISPR/Cas12a-based visual field detection system for rapid and sensitive diagnosis of major viral pathogens in calf diarrhea

**DOI:** 10.3389/fcimb.2025.1616161

**Published:** 2025-08-28

**Authors:** Junzhen Chen, Yu Wang, Rezeguli Aikebaier, Haoran Liu, Yingxin Li, Li Yang, Areayi Haiyilati, Lixia Wang, Qiang Fu, Huijun Shi

**Affiliations:** ^1^ College of Veterinary Medicine, Xinjiang Agricultural University, Ürümqi, Xinjiang, China; ^2^ Xinjiang Key Laboratory of New Drug Research and Development for Herbivores, Ürümqi, Xinjiang, China

**Keywords:** viral metagenomic sequencing, RAA-CRISPR/Cas12a, calf diarrhea, diagnostics, PCR

## Abstract

Calf diarrhea is a complex digestive disorder in cattle that imposes significant economic losses in terms of calf mortality, growth impairment, and treatment costs. Both infectious and non-infectious agents contribute to its aetiology; however, most of the infectious cases are caused by viruses, often accompanied by severe co-infections. To identify viral culprits, we performed viral metagenomic sequencing on three pooled samples from the 150 diarrheal samples from Xinjiang, China, which helped with identification of the following four predominant agents: bovine nepovirus (BNeV), bovine coronavirus (BCoV), bovine viral diarrhea virus (BVDV) and bovine enterovirus (BEV). Currently, the process of diagnosing these pathogens involves time-consuming workflows, limited sensitivity, poor portability, and lack of field applicability. Keeping these diagnostic shortcomings in mind, an integrated platform called RAA-CRISPR/Cas12a system was developed by combining recombinase-aided amplification (RAA) at 37°C with CRISPR/Cas12a-mediated fluorescence detection, which achieved 100–100,000 times higher sensitivity than conventional polymerase chain reaction (PCR) (detection limits: 1–10 copies/μL) and demonstrated 100% specificity against non-target pathogens. Clinical validation of sensitivity and specificity of 252 samples revealed 1.6–4.9 times higher detection rates (239 positives) than PCR (81 positives), which was consistent with PCR-confirmed cases. The assay’s 40-min. workflow enables rapid on-site deployment without specialized instrumentation, as it requires only a portable heat block and blue LED transilluminator. Hence, with its laboratory accuracy and field applicability, this method helps in early identification of pathogens, outbreak containment and mitigation of economic loss in the global cattle industry.

## 1 Introduction

Diarrhea is a common digestive disease that occurs year-round among calves, with a higher prevalence during the spring and winter seasons (Elgioushy et al., 2025). Affected calves typically present with clinical symptoms such as severe diarrhea, loss of appetite, mucosal congestion and lethargy. Without prompt intervention, sick calves are at risk of dehydration, emaciation and, in severe cases, mortality or growth retardation (Ji et al., 2022), posing substantial challenges to the sustainable development of the cattle industry (Dall et al., 2021). The complex pathogenesis of calf diarrhea mostly involves viral infections that are particularly problematic due to viral infections resistance to conventional control measures. Viral agents often induce prolonged or sub-clinical infections (Wei et al., 2021), which can increase the risk of secondary bacterial co-infections (Yanfei et al., 2025). Among viral pathogens, bovine nepovirus (BNeV), bovine coronavirus (BCoV), bovine viral diarrhea virus (BVDV) and bovine enterovirus (BEV) are recognized as the primary aetiological agents of viral diarrhea in calves.

BNeV is a single-stranded, positive-sense RNA virus that belongs to the genus *Nebovirus* (family Caliciviridae) (Candido et al., 2016). BCoV is an enveloped, single-stranded, positive-sense RNA virus that belongs to the genus *Coronavirus* (family Coronaviridae) (Clark, 1993). BVDV is a single-stranded, positive-sense RNA virus that belongs to the genus *Pestivirus* (family Flaviviridae) (Collett et al., 1988). However, BEV is a non-enveloped, single-stranded, positive-sense RNA virus that belongs to the genus *Enterovirus* (family Picornaviridae) (Liu et al., 2019; Doedens and Kirkegaard, 1995). Global epidemiological studies indicate that BNeV, BCoV, BVDV and BEV are widely distributed pathogens associated with calf diarrhea. Their high incidence, rapid transmission, overlapping clinical manifestations and potential for co-infections have raised significant concerns in livestock industries worldwide (Bianchi et al., 2019; Hansen et al., 2015; Hilton et al., 2006). Although multiple methods can diagnose these viruses, most of these methods rely on specialized laboratory equipment, require prolonged processing times or lack field applicability, and all these shortcomings hinder quick and accurate identification of on-site pathogens. Hence, a detection platform characterized by quick processing time and high sensitivity and specificity must be urgently developed to enable timely surveillance and control of these viral pathogens.

Recombinase-aided amplification (RAA) is a novel and rapid isothermal nucleic acid amplification technique that enables rapid target DNA amplification at 30–37°C within 42 min., and it requires no heat-stable enzymes or thermal cyclers (Barrangou et al., 2007). This method employs single-stranded DNA-binding proteins, recombinase and DNA polymerase, providing advantages such as simplified primer design, rapid amplification, high sensitivity and minimal equipment dependency (Wang et al., 2020). CRISPR-associated (Cas) proteins, including Cas12, Cas13 and Cas14, are nucleases that can bind to and cleave specific DNA sequences. These proteins exhibit both cis-cleavage (target-specific) and trans-cleavage (collateral non-specific) activities (Arefian et al., 2011; Moltzahn et al., 2011; Robinson et al., 2017). Notably, Cas12 proteins have emerged as essential tools for detecting nucleic acids due to their robust target recognition and collateral cleavage properties, which facilitate signal amplification. The integration of Förster resonance energy transfer (FRET)–based reporters, which are fluorescently labelled single-stranded DNA probes paired with quenchers, allows real-time, visual detection of target nucleic acids. Recent advances have harnessed RAA-CRISPR/Cas technology for pathogen detection. For instance, Kellner et al. (2019) demonstrated multiplex discrimination of Zika and Dengue viruses using Cas12, while Sima et al. (2022) utilized this platform for field diagnosis of human African trypanosomiasis. Similarly, Zhang et al. (2024) achieved high-risk human papillomavirus (HPV) genotyping (types 16 and 18) with Cas12-mediated specificity. Based on these innovations, this study aimed to develop a rapid, sensitive and field-deployable RAA-CRISPR/Cas12a assay for detecting major viral pathogens causing calf diarrhea and addressing critical gaps in point-of-care diagnostics for calf diarrhea simultaneously.

## 2 Materials and methods

### 2.1 Sample collection and preparation

For this study, 150 diarrhea samples were obtained from Maigaiti and Hutubi counties in Xinjiang, China, and categorized them into three samples for viral metagenome sequencing based on the time and place of collection. For clinical validation, 252 clinical samples were collected from different cattle farms in Makit, Hutubi, Urumqi, Changji and Bole Counties, Xinjiang, China. All the samples were collected in the form of anal swabs from calves with clinically suspected diarrhea. Ethical approval for this study was obtained from the Laboratory Animal Welfare and Ethics Committee of Xinjiang Agricultural University (Approval No. 2022038). All samples were immediately homogenized in DEPC-treated, RNase-free phosphate-buffered saline (PBS, pH 7.4) at a 1:5 (w/v) ratio, vortexed at 3,000 rpm for 2 min. and stored at −80°C.

### 2.2 Viral macro-sequencing

After homogenization in PBS, samples were submitted to Shanghai Tanpu Biotechnology Co., Ltd (Shanghai, China) for viral metagenomic sequencing. Genomic DNA and RNA were extracted using the Viral DNA Kit (Solarbio, China, Cat. No. D3892 and Invitrogen, USA; Cat. No. 15596026) according to the manufacturer’s protocol. Genomic DNA was sheared into 300~500 bp fragments by Covaris M220 ultra-sonicator (peak incident power: 50 W, duty factor: 20%, cycles per burst: 200). RNA was fragmented in Mg^2+^-containing buffer (94°C, 5 min.) and reverse transcribed into cDNA using RT Easy™ II Kit (Foregene, China; Cat. No. RT-01022). DNA and cDNA libraries were prepared with the Illumina TruSeq Nano DNA/RNA Library Prep Kit and quantified via Qubit 4.0. Libraries were sequenced on an Illumina NovaSeq 6000 platform (PE150 mode), which generated approximately 20 Gb raw data per sample. Raw reads were filtered using Fastp v0.20.0 (with the following parameters: remove reads with Q-score < 20, length < 50 bp or adapter contamination), and reads that aligned to the bovine genome (ARS-UCD1.2) were removed using BBmap v38.51. Clean reads were then classified via Kraken2 v2.1.2 with the Standard Database and Bracken v2.6.2 estimated species abundance. Finally, raw viral metagenomic sequencing read archive were submitted to the National Center for Biotechnology Information (NCBI) Sequence Read Archive (SRA) repository under project number PRJNA1244845.

### 2.3 Construction of BNeV, BVDV, BEV and BCoV standard plasmids

The nucleotide sequences of the RNA-dependent RNA polymerase (*RdRp*) gene of BNeV (GenBank accession no. MN607031.1), nucleocapsid (*N*) gene of BCoV(LC494175.1), 5’ untranslated region (*5’UTR*) of BVDV (AJ133738.1) and viral protein 1 (*VP1*) gene of BEV (D00214.1) were synthesized by Beijing Tsingke Biotechnology Co. Ltd. (China). Each target sequence was cloned into the pUC57 plasmid vector using *Eco*R I and *Hin*d III restriction sites (**Supplementary Figure 1S**). The recombinant plasmids were transformed into *Escherichia coli* DH5α competent cells, purified using the Plasmid Mini Kit (Qiagen, Germany) and quantified via Qubit 4.0 Fluorometer (Thermo Fisher Scientific, USA). Sanger sequencing confirmed 100% identity to the reference sequences. Aliquots were stored at −20°C as quantification standards for downstream assays.

### 2.4 Establishment and optimization of RAA reactions

Along with crRNAs targeting protospacer adjacent motif (PAM) sites, polymerase chain reaction (PCR)- and RAA-specific primers were designed based on conserved sequences of the BNeV *RdRp*, BCoV *N*, BVDV *5’UTR* and BEV *VP1* genes (**Table 1**), and RAA reactions were performed using the RAA Nucleic Acid Amplification Kit (Qitian, China, Cat. No. QT-RAA-100) in a 25.0 μL system containing the following: 12.5 μL reaction buffer, 1.0 μL forward/reverse primers (10.0 μM each), 2.5 μL magnesium acetate (280.0 mM), 1.0 μL DNA template and 7.0 μL nuclease-free ddH_2_O. Amplification products were analyzed using 2% agarose gel electrophoresis (100 V, 30 min.) and visualized using a Gel DocTM XR+ System (Bio-Rad, USA). Standard plasmids (1×10^6^ copies/μL) were amplified at 34°C, 37°C, 40°C and 43°C for 30 min. to determine the optimal reaction temperature. Then, under the optimal temperature, reactions were allowed to occur for 10, 15, 20 and 30 min. to identify the minimal required duration. For comparison, conventional RT-PCR assays were established using PrimeSTAR Max DNA Polymerase (Takara, Japan) under the following conditions: 95°C for 5 min.; 35 cycles of 95°C for 30 s, 56–60°C for 30 s, 72°C for 30 s and final extension at 72°C for 5 min.

**Table 1:** The primers and crRNA sequences.

Assay	Name	Oligonucleotide sequences (5’→3’)
PCR	BNeV-P-F	CTGAAACCAGAACCATCCAA
	BNeV-P-R	TAACTAAGGCCGAAACATGG
	BCoV-P-F	GAAATGTTAAAACTTGGAACTAGTGATCCACA
	BCoV-P-R	CAACTCTAATCTTGATCCAAAGAAAAACGCAC
	BVDV-P-F	CCTAGCCATGCCCTTAGTAGGACT
	BVDV-P-R	GGAACTCCATGTGCCATGTACA
	BEV-P-F	CCAATGCGTATTCCACGGTTTAT
	BEV-P-R	TCTTCTTGTATGGTGCTTGTCTG
RAA	BNeV-R-F	CAKGTKKCRGCCYTAGTTMWYAAATCT
	BNeV-R-R	TCCTTCTCTAATTAAATCACTATTGCWYGG
	BCoV-R-F	GAGAAATGTTAAAACTTGGAACTAGTGATCC
	BCoV-R-R	CCAACTCTAATCTTGATCCAAAGAAAAACG
	BVDV-R-F	CAAAGCACATCTTAACCTGAGCGGGGGTCG
	BVDV-R-R	CAGCAGAGATTTTTAGTAGCAATACAGTGGG
	BEV-R-F	AATGTTACCACCGAGCAGCACACCACTTACCA
	BEV-R-R	GAGCTCATGAAGGGTACTGAGAATTGGGCAGG
Cas12-based detection	BNeV-crRNA1	UAAUUUCUACUAAGUGUAGAUACCAUGUUUCGGCCUUAGUUAUC
	BNeV-crRNA2	UAAUUUCUACUAAGUGUAGAUGGCCUUAGUUAUCAAAUCUGCUG
	BCoV-crRNA	UAAUUUCUACUAAGUGUAGAUUUUGGAUCAAGAUUAGAGUUGG
	BVDV-crRNA	UAAUUUCUACUAAGUGUAGAUACCGACUGUUACGAAUACAGCCU
	BEV-crRNA1	UAAUUUCUACUAAGUGUAGAUUGAUGGGUACGCACGAUUCAUGG
	BEV-crRNA2	UAAUUUCUACUAAGUGUAGAUAGUGGCAAUCUGGGUGCAACCCC

### 2.5 Establishment and optimization of the RAA-CRISPR/Cas12a assay

**Figure 1:**
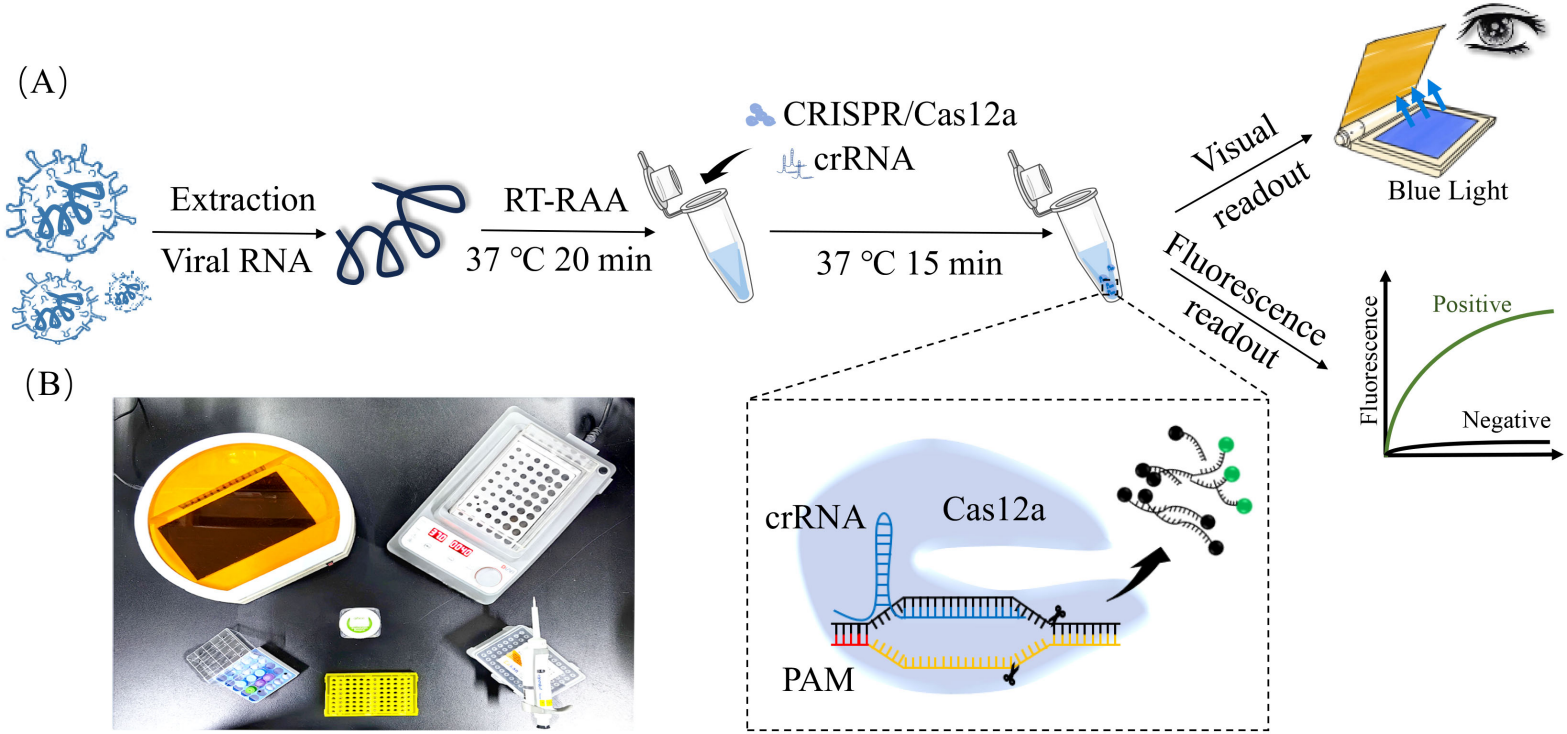
RAA-CRISPR/Cas12a-Based detection of major viral pathogens in calf diarrhea. **(A)** Flowchart of RAA-CRISPR/Cas12a assay. The viral RNA was isolated from clinical sample, and RNA was reverse transcribed into cDNA. RAA amplification was performed at 37°C for 20 min. CRISPR/Cas12a detection reaction was incubated at 37°C for 15 min. Fluorescence signals were observed under a blue LED transilluminator and documented using a gel system. Positive samples exhibited bright green fluorescence, while negatives remained dark. **(B)** Minimum equipment needed to run protocol. With appropriate biosafety level 2 requirements, the minimum equipment required to run the protocol included Eppendorf tubes with reagents, portable heat block or water bath (37°C), nuclease-free water, pipettes, tips and blue LED transilluminator.

The CRISPR/Cas12a detection system was established as follows: a 30 μL reaction mixture contained 3 μL 10 × NEB Buffer 2.1 (New England Biolabs, USA; Cat. No. B7202S), 1.5 μL Cas12a nuclease (1 μM; New England Biolabs, USA, Cat. No. M0653T), 1.5 μL crRNA (200 nM; synthesized by Tsingke Biotechnology, China), 1.5 μL fluorescent reporter (FAM-ddT-ssDNA-BHQ1, 500 nM; Tsingke Biotechnology Co., Ltd.), 4 μL RAA amplification product (template) and 18.5 μL nuclease-free ddH_2_O. The mixture was incubated at 37°C for 15 min. in a thermal cycler (Bio-Rad, USA) that was protected from light. Fluorescence signals were visualized using a blue LED transilluminator (Azure Biosystems, USA), and they were quantified with ImageJ v1.53 (NIH, USA) (**Figure 1**). For optimization of Cas12a nuclease concentration, reactions were performed with Cas12a at 100, 200, 400, 600 and 800 nM (crRNA fixed at 200 nM). For optimization of crRNA concentration, crRNA was evaluated at 100–450 nM (in 50 nM increments) with Cas12a fixed at 200 nM. All reactions included negative controls (nuclease-free ddH_2_O) and positive controls (1 × 10^6^ copies/μL standard plasmid).

### 2.6 Specificity, sensitivity and reproducibility testing

#### 2.6.1 Specificity

To evaluate assay specificity, the RAA-CRISPR/Cas12a system was evaluated against genomic DNA/cDNA from BNeV, BVDV, BEV, BCoV, *E. coli* (ATCC 25922) and *Salmonella enterica* (ATCC 14028), with nuclease-free water as a negative control. Reactions were performed in triplicate under optimized conditions.

#### 2.6.2 Sensitivity

Ten times serial dilutions (1 × 10^6^ copies/μL (1×10^7^ copies/μL − 1 × 10^0^ copies/μL) of BNeV, BCoV, BVDV and BEV standard plasmids were tested to determine the lower limit of RAA-CRISPR/Cas12a assay detection. Each dilution was analyzed in triplicate.

#### 2.6.3 Reproducibility

For intra-batch reproducibility testing, three plasmid concentrations (5 × 10^6^, 5 × 10^5^ and 5 × 10^4^ copies/μL) were evaluated in triplicate within the same run. For inter-batch reproducibility testing, the same concentrations were analyzed across three independent experiments performed on different time. The coefficient of variation (CV) was calculated as follows: CV (%) = (standard deviation/mean) × 100.

### 2.7 Clinical sample validation

Total RNA was extracted from clinical samples using TRIzol™ Reagent according to the manufacturer’s protocol. RNA concentration and purity were determined using a NanoDrop 2000 spectrophotometer (Thermo Fisher Scientific, USA). RNA (1 μg) was reverse transcribed into cDNA using the PrimeScript™ RT Reagent Kit (Takara, Japan) with random hexamer primers under the following conditions: 37°C for 15 min., 85°C for 5 s. Furthermore, 252 clinical samples collected from Xinjiang, China, were analyzed simultaneously using both the established RAA-CRISPR/Cas12a assay and conventional PCR.

### 2.8 Statistical analysis

Statistical analyses were performed using SPSS 17.0 software (IBM Corp., Armonk, NY, USA). Continuous variables are presented as mean ± standard deviation (SD), depending on data normality assessed by the student *t*-test, and statistical significance was defined as *P* < 0.05, with asterisks denoting the following: Note: *, *P* < 0.05; **, *P* < 0.01; ***, *P* < 0.001.

## 3 Results

### 3.1 Viral macro-genome sequencing

**Figure 2:**
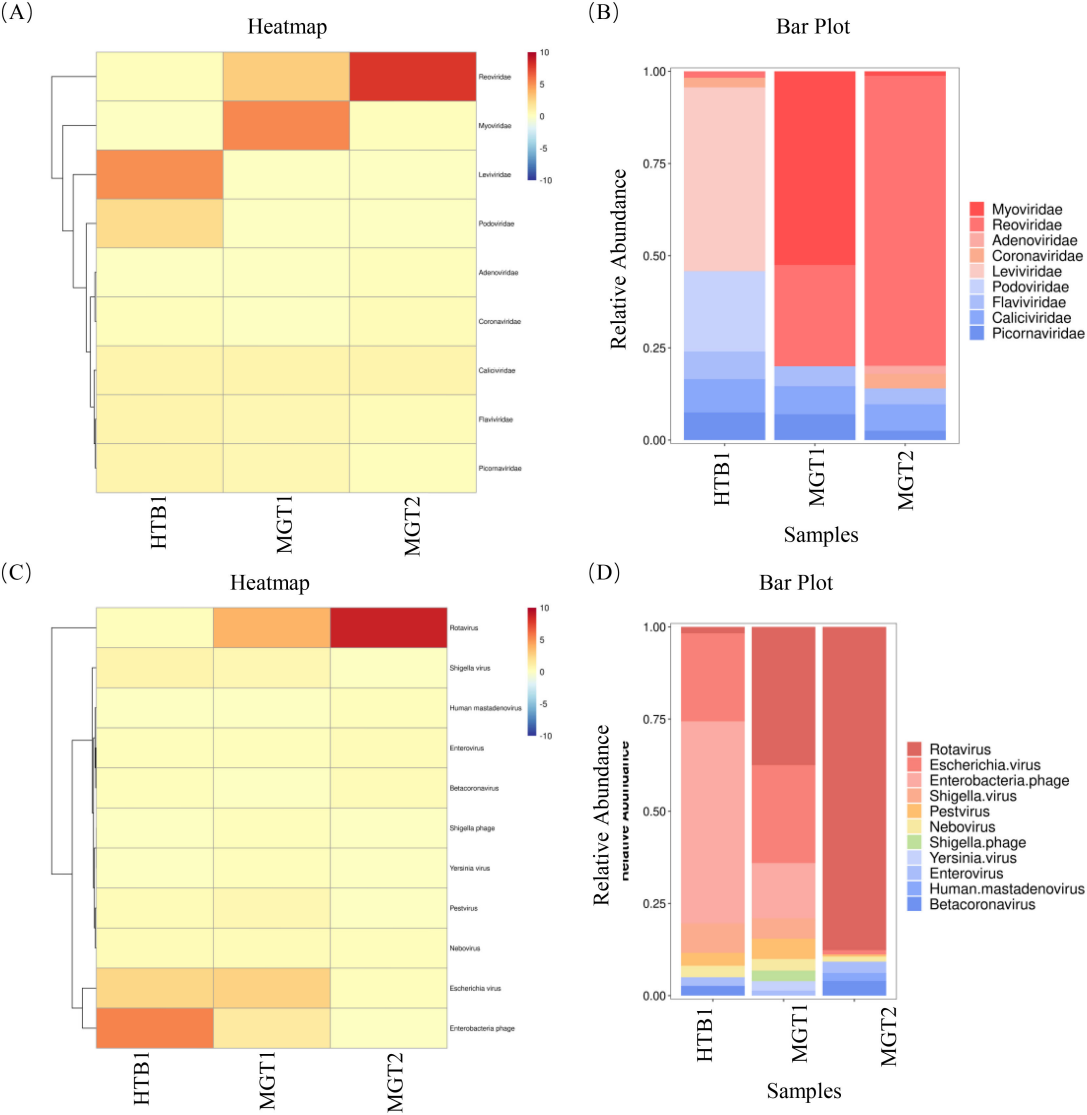
The results of viral macro-genome sequencing assembly. **(A, B)** Nine viral families were identified across the three samples, with Reoviridae, Flaviviridae, Caliciviridae and Picornaviridae, which were consistently detected in all samples. **(C, D)** taxonomic annotation resolved 11 viral genera, including *Rotavirus*, *Escherichia virus*, *Pestivirus*, *Nebovirus* and *Enterovirus*, which were ubiquitously present in all samples.

The clean reads were assembled *de novo* using SPAdes (v3.14.1). Contigs ≥1,000 bp were retained for further analysis (**Table 2**). Assembly statistics revealed 46,176 contigs ≥1,000 bp, including 3,273 (≥5,000 bp), 946 (≥10,000 bp), 151 (≥25,000 bp), and 38 (≥50,000 bp). Viral contigs were annotated using NCBI nt via BLAST+ with an E-value cutoff of ≤1E-5. Across all samples, the following nine viral families were identified: Myoviridae, Reoviridae, Adenoviridae, Coronaviridae, Leviviridae, Podoviridae, Flaviviridae, Caliciviridae and Picornaviridae (**Figures 2A, B**). Furthermore, taxonomic classification resolved the following 11 genera: *Rotavirus*, *Escherichia virus*, *Enterobacteria phage*, *Shigella virus*, *Pestivirus*, *Nebovirus*, *Shigella phage*, *Yersinia virus*, *Enterovirus*, *Human mastadenovirus and Betacoronavirus* (**Figures 2C, D**).

**Table 2:** Result data of sample genome assembly.

Name	Contigs length (bp)					
	≥1,000	≥5,000	≥10,000	≥25,000	≥50,000	Largest contig
HTB1	45363	3237	935	147	36	142989
MGT1	813	23	8	3	2	67646
MGT2	1593	13	3	1	0	36356
add up the total	46176	3273	946	151	38	—

### 3.2 Establishment and optimization of RAA assay

**Figure 3:**
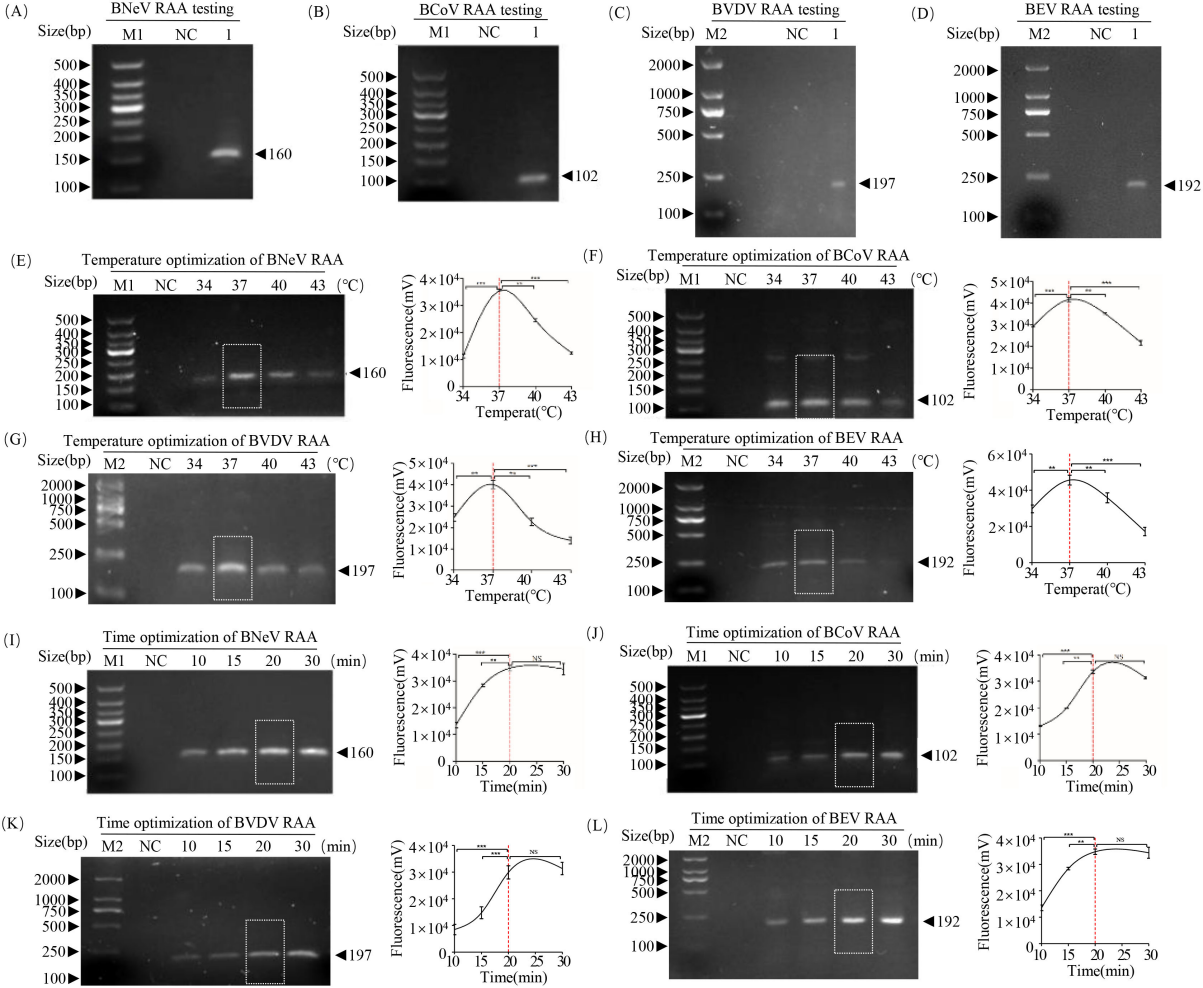
Establishment and optimization of RAA assays for BNeV, BCoV, BVDV and BEV. **(A–D)** Agarose gel (2%) electrophoresis of RAA products targeting **(A)** BNeV (160 bp, **(B)** BCoV (102 bp), **(C)** BVDV (197 bp) and **(D)** BEV (192 bp). **(E–H)** Temperature optimization (34°C, 37°C, 40°C, 43°C; 30 min.) for **(E)** BNeV, **(F)** BCoV, **(G)** BVDV and **(H)** BEV. Grayscale intensity (ImageJ) of bands was quantified and normalized to the 37°C group (dashed line). **(I–L)** Time course analysis (10, 15, 20, 30 min.; 37°C) for **(I)** BNeV, **(J)** BCoV, **(K)** BVDV and **(L)** BEV. Significant differences in grayscale values are indicated. **P < 0.01 vs. 15 min.; NS, not significant vs. 30 min.; Student’s *t*-test.

Using recombinant plasmids of BNeV, BCoV, BVDV and BEV as templates, RAA isothermal amplification generated specific bands of 160 bp (BNeV), 102 bp (BCoV), 197 bp (BVDV) and 192 bp (BEV), which were consistent with expected sizes (**Figures 3A–D**). For temperature optimization, reactions performed at 34–43°C revealed that 37°C yielded the highest amplification efficiency for all four viruses, and their band intensity declined at higher temperatures (**Figures 3E–H**). For time course optimization, detectable amplification occurred at 37°C within 10 min., and fluorescence signals peaked at 20 min. (**Figures 3I–L**). Grayscale analysis showed significant signal enhancement at 20 rather than 15 min. (*P* < 0.05); however, no such difference was observed between 20 and 30 min. Thus, 20 min. was selected as the optimal reaction time.

### 3.3 Establishment and optimization of the RAA-CRISPR/Cas12a assay

**Figure 4:**
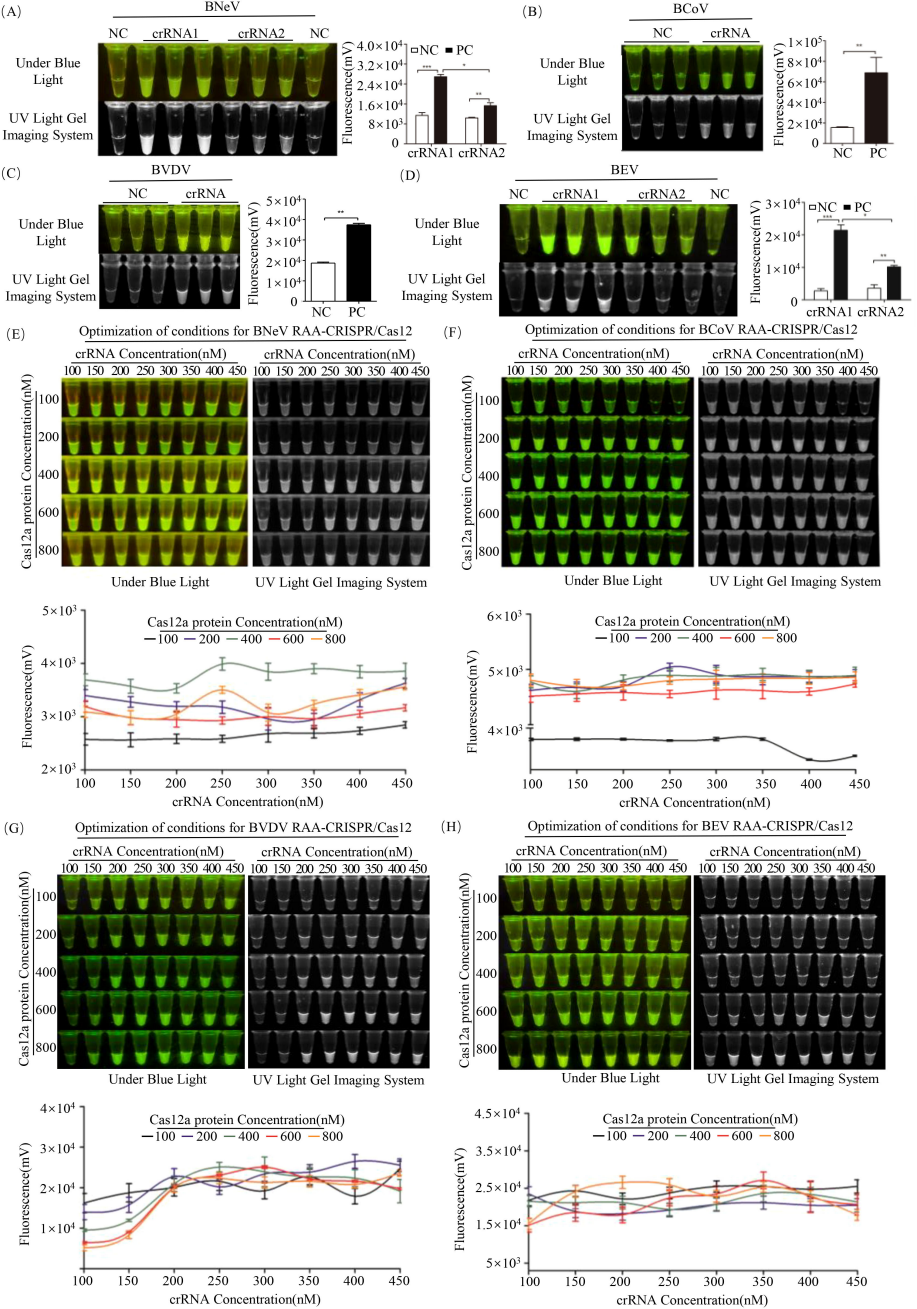
Optimization of the RAA-CRISPR/Cas12a assay for BNeV, BCoV, BVDV and BEV. **(A-D)** crRNA screening. Fluorescence intensity (grayscale analysis, ImageJ) of candidate crRNAs (crRNA1 and crRNA2) for each virus. **(E-H)** Optimization of Cas12a and crRNA concentrations. Fluorescence intensity (grayscale analysis, ImageJ) under varying crRNA (100–450 nM) and Cas12a (100–800 nM) concentrations. *P < 0.05; **P < 0.01; ***P < 0.001.

To maximize collateral cleavage activity of Cas12a, two candidate crRNAs targeting conserved regions of BNeV and BEV were evaluated. crRNA1 for both viruses exhibited significantly higher fluorescence intensity than crRNA2 (*P* < 0.01, Student’s *t*-test; **Figures 4A, D**), indicating superior target recognition and trans-cleavage efficiency. Similarly, crRNAs for BCoV and BVDV displayed distinct fluorescence signals compared to negative controls (**Figures 4B, C**), confirming their functionality.

Gradient testing revealed pathogen-specific optimal concentrations as follows: BNeV: 400 nM Cas12a + 250 nM crRNA (**Figure 4E**), BCoV: 200 nM Cas12a + 250 nM crRNA (**Figure 4F**), BVDV: 600 nM Cas12a + 300 nM crRNA (**Figure 4G**) and BEV: 800 nM Cas12a + 200 nM crRNA (**Figure 4H**). These concentrations were chosen for subsequent assays to maximize detection sensitivity.

### 3.4 Specificity of the RAA-CRISPR/Cas12a assay

**Figure 5:**
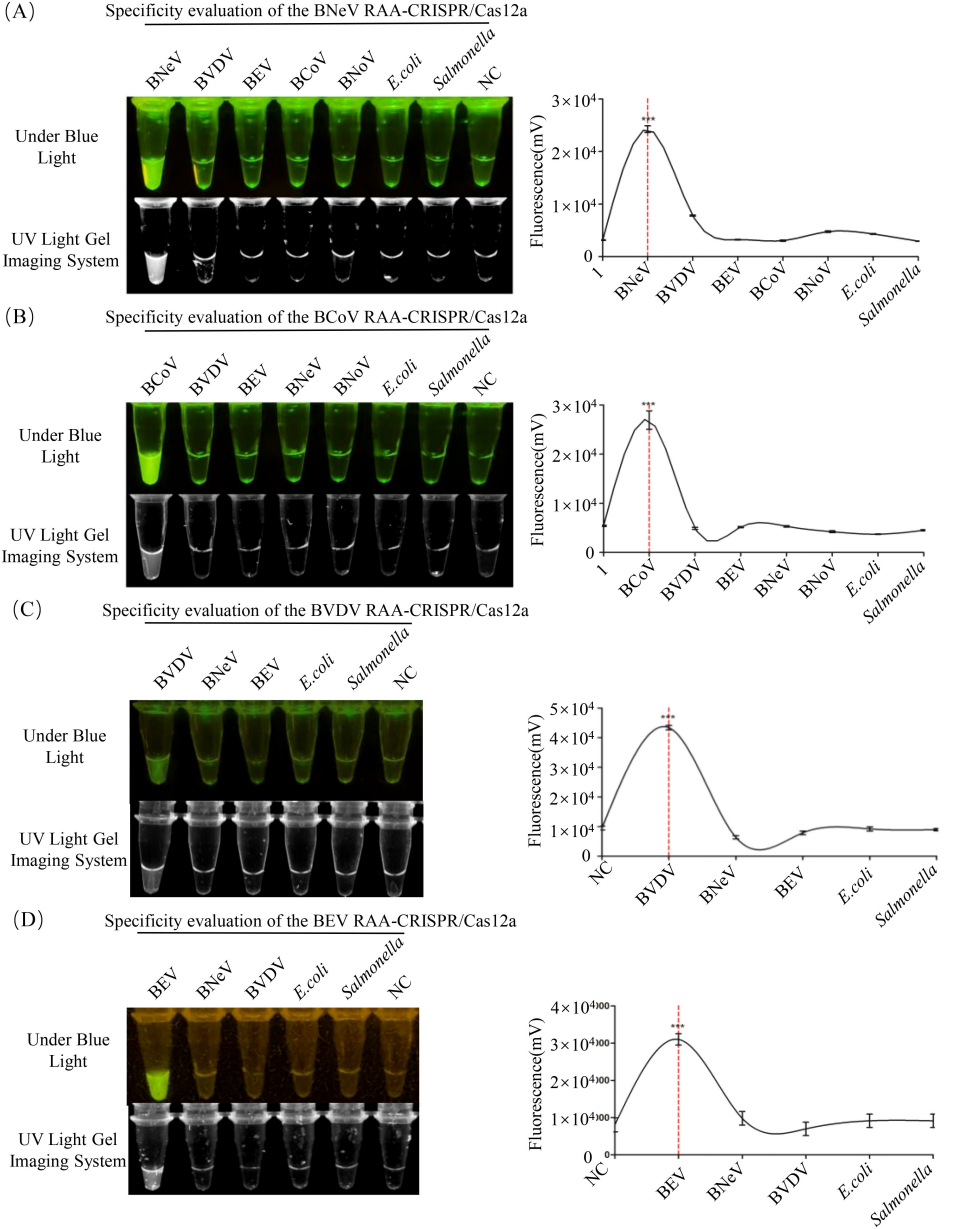
Specificity evaluation of the RAA-CRISPR/Cas12a assay. Fluorescence signals were assessed using cDNA from **(A)** BNeV, **(B)** BCoV, **(C)** BVDV and **(D)** BEV, genomic DNA from *E*. *coli* and *S. enterica*, and nuclease-free water (negative control, NC). Reactions were performed under optimized conditions (37°C, 20 min.). Images were captured under blue LED transilluminator illumination, and fluorescence intensity was quantified using ImageJ software.

To evaluate the specificity of the RAA-CRISPR/Cas12a and PCR assays for BNeV, BCoV, BVDV and BEV, each system was evaluated against cDNA/DNA from the target viruses, *E. coli*, *S*. *enterica* and nuclease-free water (negative control). In the RAA-CRISPR/Cas12a system, fluorescence signals were observed exclusively for the target viruses (**Figures 5A–D**). Grayscale analysis (ImageJ) confirmed that fluorescence intensities for BNeV, BCoV, BVDV and BEV were significantly higher than those of non-target pathogens and negative controls (*P* < 0.001, Student’s *t*-test; **Figures 5A–D**). No cross-reactivity was detected with *E. coli*, *S. enterica* or negative controls. In the PCR detection system, agarose gel electrophoresis demonstrated amplification bands exclusively for the target viruses (**Supplementary Figure 2S**), with no detectable products for non-target pathogens or negative controls. These results confirmed that both assays exhibit high specificity for their respective targets, showing no cross-reactivity to phylogenetically unrelated pathogens or bacterial DNA.

### 3.5 Sensitivity of the RAA-CRISPR/Cas12a assay

**Figure 6:**
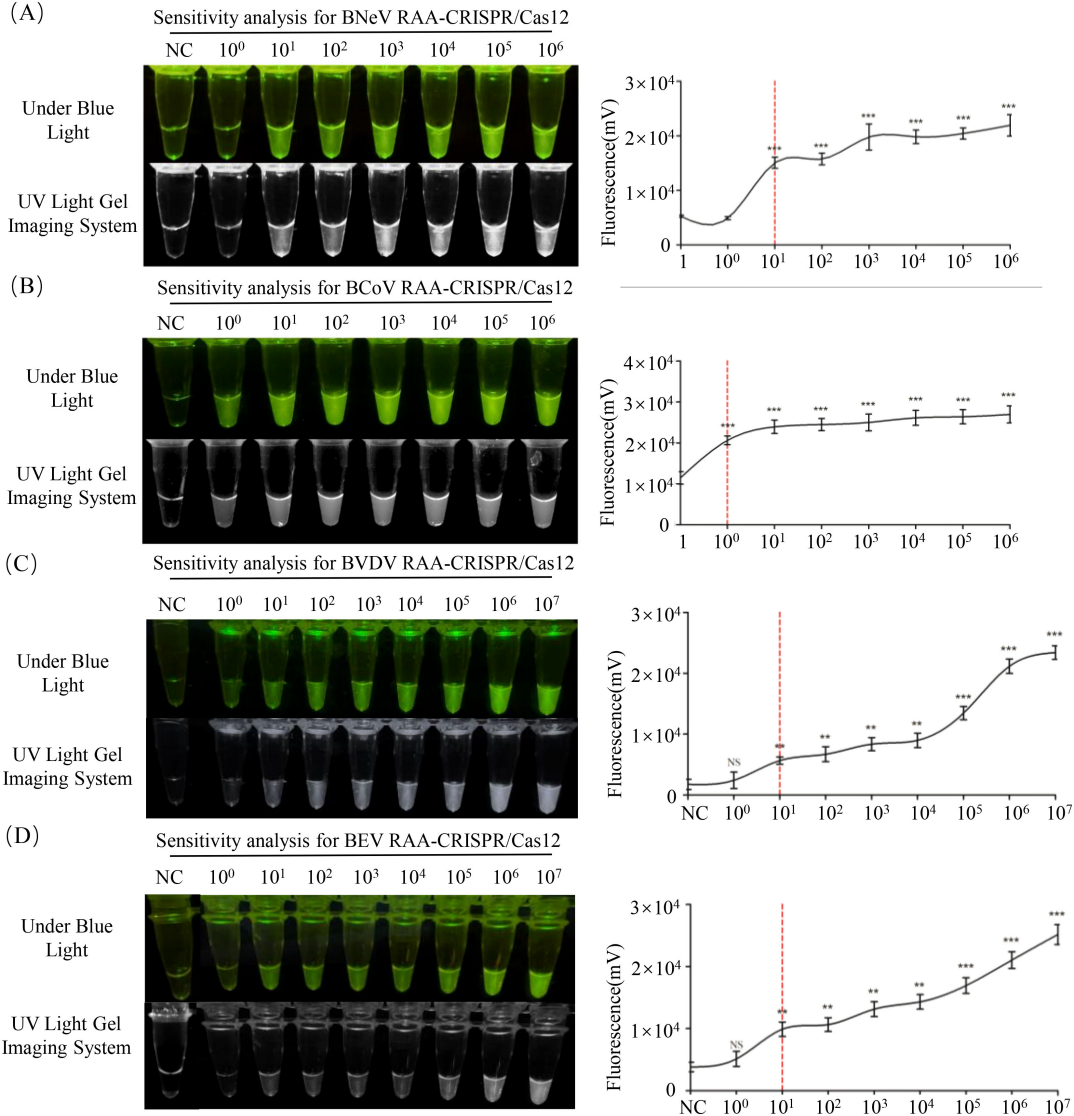
Sensitivity analysis of RAA-CRISPR/Cas12a assays. **(A–D)** Detection limits for **(A)** BNeV, **(B)** BCoV, **(C)** BVDV and **(D)** BEV. Serial dilutions that were 10 times of standard plasmids were used as templates, ranging from 1 × 10^6^ copies/μL (1 × 10^7^ copies/μL) − 1 × 10^0^ copies/μL. Fluorescence signals were captured using a blue LED transilluminator and quantified via ImageJ (grayscale analysis). *P < 0.05; **P < 0.01; ***P < 0.001; NS, non-significant.

The detection limits of the RAA-CRISPR/Cas12a and PCR assays were assessed using serial dilutions that are 10 times of standard plasmids. Fluorescence signals (grayscale analysis, *P* < 0.01 vs. negative control; Student’s *t*-test) were detectable at 10 copies/μL in the RAA-CRISPR/Cas12a system for BNeV, BVDV and BEV (**Figures 6A, C, D**). Significant fluorescence (*P* < 0.001) was observed at a concentration of 1 copy/μL in the RAA-CRISPR/Cas12a system for BCoV (**Figure 6B**). In contrast, the detection limits for conventional PCR were 1 × 10^5^ copies/μL for BNeV and BCoV and 1 × 10^3^ copies/μL for BVDV and BEV (**Supplementary Figure 3S**). The RAA-CRISPR/Cas12a assay demonstrated sensitivity that was 100–100,000 times greater than conventional PCR, with the most significant improvements observed for BCoV (100,000 times) and BNeV (10,000 times).

### 3.6 Repeatability of the RAA-CRISPR/Cas12a assay

To evaluate assay reproducibility, intra- and inter-batch variability were assessed using plasmid standards at concentrations of 5 × 10^6^, 5 × 10^5^ and 5 × 10^4^ copies/μL. Intra-batch reproducibility was assessed through triplicate testing within a single run, while inter-batch reproducibility was evaluated by conducting three independent experiments on different days. For BNeV, the intra-batch CVs ranged from 2.16% to 3.35%, while the inter-batch CVs ranged from 1.41% to 2.06% across different concentrations. BCoV exhibited intra-batch CVs of 0.64–3.48% and inter-batch CVs of 1.41–4.88%. For BVDV, intra-batch and inter-batch coefficients of variation (CVs) were 2.21–2.87% and 2.29–3.40%, respectively. BEV demonstrated intra-batch CVs of 2.47–3.15% and inter-batch CVs of 3.41–3.87% (**Table 3**). No fluorescence signals were observed in the negative controls. These results confirmed that the RAA-CRISPR/Cas12a system is highly reproducible and stable for all four targets.

**Table 3:** Repeatability of the RAA-CRISPR/Cas12a assay.

Viruses	Plasmid concentration (copies/μL)	coefficient of variation (CV%)	
		Intra-batch CV	Inter-batch CV
BNeV	5 × 10^4^	2.16	2.06
	5 × 10^5^	2.91	1.87
	5 × 10^6^	3.35	1.41
BCoV	5 × 10^4^	2.01	2.25
	5 × 10^5^	0.64	1.41
	5 × 10^6^	3.48	4.88
BVDV	5 × 10^4^	2.21	2.29
	5 × 10^5^	2.87	3.10
	5 × 10^6^	2.86	3.40
BEV	5 × 10^4^	2.47	3.58
	5 × 10^5^	3.15	3.87
	5 × 10^6^	2.93	3.41

### 3.7 Clinical sample validations

**Figure 7:**
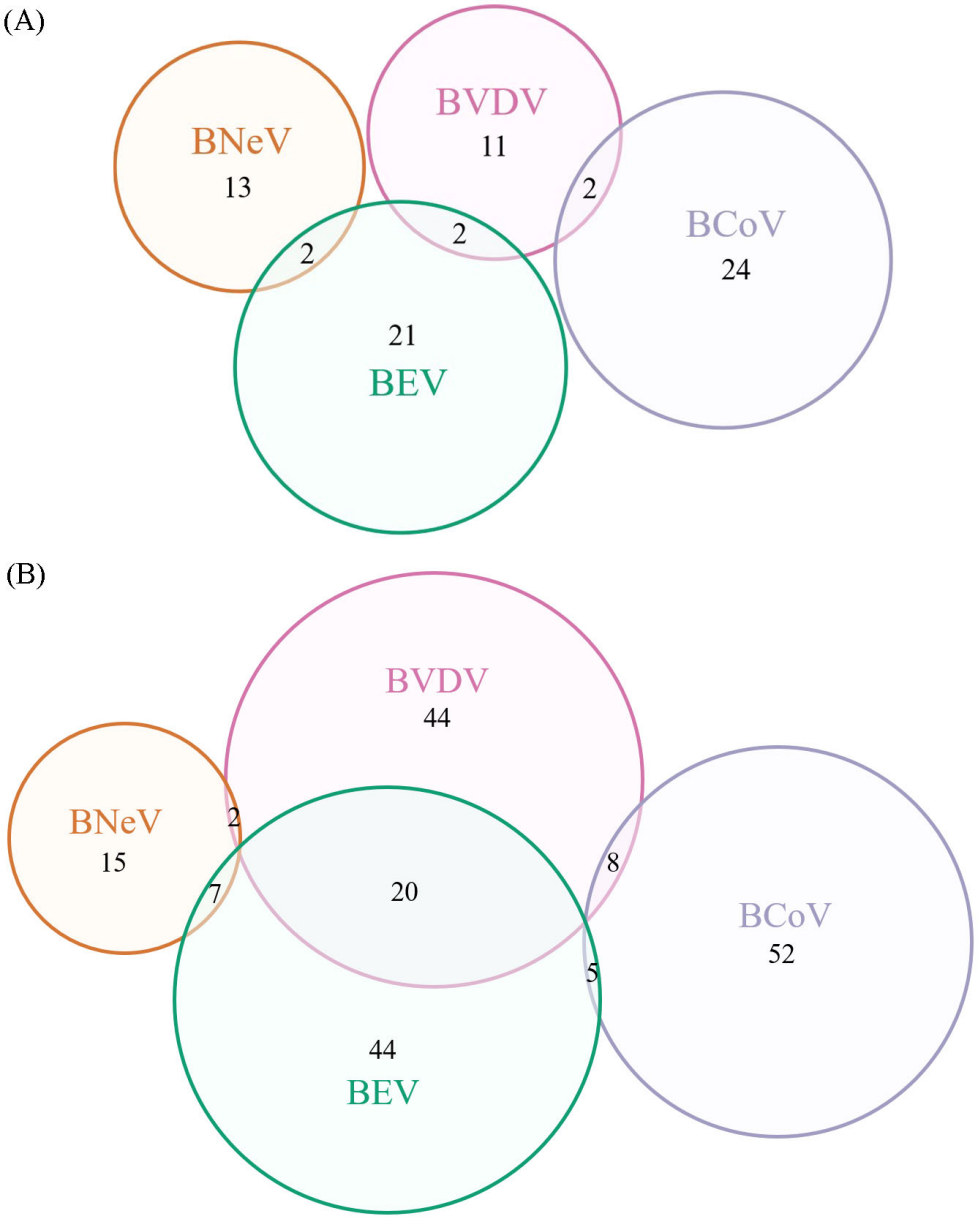
Comparative clinical detection of calf diarrhea pathogens. **(A)** RT-PCR results: BNeV (15/252, 5.95%), BCoV (26/252, 10.32%), BVDV (15/252, 5.95%) and BEV (25/252, 9.92%). **(B)** RAA-CRISPR/Cas12a results: BNeV (24/252, 9.52%), BCoV (65/252, 25.79%), BVDV (74/252, 29.37%) and BEV (76/252, 30.16%). Red asterisks indicate statistically significant differences between methods. ***P < 0.001; Student’s *t*-test.

The diagnostic performance of RAA-CRISPR/Cas12a was evaluated using 252 clinical anal swab samples and compared to conventional PCR. As shown in **Table 4**, the RAA-CRISPR/Cas12a assay demonstrated significantly higher positive detection rates for all four pathogens compared to PCR: BNeV (9.52% vs. 5.95%), BCoV (25.79% vs. 10.32%), BVDV (29.37% vs. 5.95%), and BEV (30.16% vs. 9.92%). Statistical analysis revealed that the RAA-CRISPR/Cas12a system exhibited 1.6 to 4.9 times higher sensitivity than PCR across all targets (**Figures 7A, B**, **Supplementary Figure 4S**). Notably, all PCR-positive samples (*n* = 81) were consistently identified by the RAA-CRISPR/Cas12a method, thereby confirming 100% specificity for true positives.

**Table 4:** Results of PCR and RAA-CRISPR/Cas12a clinical assays.

Virus	PCR positivity (%)	RAA-CRISPR/Cas12a positivity (%)	Sensitivity (fold)
BNeV	5.95% (15/252)	9.52% (24/252)	1.60
BCoV	10.32% (26/252)	25.79% (65/252)	2.50
BVDV	5.95% (15/252)	29.37% (74/252)	4.93
BEV	9.92% (25/252)	30.16% (76/252)	3.04
BNeV and BVDV	0% (0/252)	0.79% (2/252)	–
BNeV and BEV	0.79% (2/252)	2.78% (7/252)	3.50
BVDV and BEV	0.79% (2/252)	7.94% (20/252)	10.00
BCoV and BVDV	0.79% (2/252)	3.17% (8/252)	4.00
BCoV and BEV	0% (0/252)	1.98% (5/252)	–

## 4 Discussion

Calf diarrhea poses a critical threat to global cattle production (Odagiri et al., 2020; Cho and Yoon, 2014), and viral pathogens account for over 70% of infectious cases along with frequent co-infections that exacerbate disease severity (Ji et al., 2024; Chen et al., 2022; Liyun et al., 2023). In this study, viral meta-genomics data from Xinjiang identified BNeV, BCoV, BVDV and BEV as dominant etiological agents, which is consistent with global epidemiological trends. Although traditional methods such as ELISA and PCR are the most recognized clinical assays, their limitations in terms of sensitivity, turnaround time and field applicability hamper the rapid management of outbreaks (Yuming et al., 2022; He et al., 2025; Bustin, 2005). For instance, the PCR assays developed this study exhibited detection limits of 10^5^ copies/μL for BNeV/BCoV and 10^3^ copies/μL for BVDV/BEV (**Supplementary Figure S3**), which are comparable to earlier multiplex PCR systems (Xiaofei et al., 2021; Ruixue et al., 2024). However, their reliance on thermal cyclers (>2 h runtime) and post-amplification gel electrophoresis limits utility in resource-constrained settings.

To address these gaps, RAA was integrated with CRISPR/Cas12a-mediated fluorescence detection in this study. This constructive collaboration allowed isothermal amplification at 37°C within 20 min. Cas12a then cleaved the FAM-BHQ1 reporter, incidentally enabling visual endpoint readout (**Figure 1**). The RAA-CRISPR/Cas12a platform achieved 100–10,000 times higher sensitivity than that of PCR, detecting as few as 1 copy/μL for BCoV and 10 copies/μL for BNeV, BVDV and BEV (**Figure 6**). Such performance surpassed recent CRISPR-based veterinary diagnostics, including porcine circovirus assays (LOD: 10 copies/μL) (Lejia, 2022), and matched clinical needs for early-stage infections. Importantly, the system had 100% specificity for non-target pathogens (**Figure 5**), which eliminated false positives that are common in serologic testing (Yuming et al., 2022). Clinical validation with 252 field samples further helped understand its superiority. RAA-CRISPR/Cas12a detected 1.6–4.9 times more positives than PCR across all targets (**Table 4**), with full concordance for PCR-confirmed cases. The 40-min. workflow that required only a portable heat block and blue LED transilluminator reduced reliance on centralized laboratories, making it ideal for point-of-care deployment.

Nevertheless, the following two limitations warranted attention: (1) RNA targets require an additional 15-min. reverse transcription step, and (2) multiplexing capabilities remain unexplored. Future work should optimize a multiplex RAA-CRISPR panel for simultaneous pathogen discrimination to further enhance field utility.

## 5 Conclusion

Calf diarrhea poses a significant threat to global cattle industries, and viral pathogens often drive outbreaks and co-infections. This study identified BNeV, BCoV, BVDV and BEV as the predominant viral agents in diarrheic calves across Xinjiang through viral metagenomic analysis. To address the limitations of conventional diagnostics, an RAA-CRISPR/Cas12a platform was developed that integrated isothermal amplification with CRISPR-based fluorescence detection. The assay demonstrated exceptional sensitivity (1–10 copies/μL), surpassing conventional PCR by 100–100,000 times, and achieved 100% specificity against non-target pathogens. Its 40-min. workflow that required only a portable heat block and blue LED transilluminator enabled on-site deployment without specialized instrumentation. Clinical validation of the method on 252 field samples showed a 1.6–4.9 times higher detection rate compared to PCR, confirming its excellent diagnostic performance. This innovation marks a key step in reducing economic losses and improving herd health management in cattle production systems worldwide.

## Data Availability

The datasets presented in this study can be found in online repositories. The names of the repository/repositories and accession number(s) can be found below: https://www.ncbi.nlm.nih.gov/, PRJNA1244845.
