# Supplementary material for: RAA-CRISPR/Cas12a-based visual field detection system for rapid and sensitive diagnosis of major viral pathogens in calf diarrhea
Source: Front Cell Infect Microbiol. 2025 Aug 28;15:1616161. doi: 10.3389/fcimb.2025.1616161 (PMC12424140; doi:10.3389/fcimb.2025.1616161)
Supplement: Supplementary file 1 [file DataSheet1.docx]

Supplementary Material

RAA-CRISPR/Cas12a-Based Visual Field Detection System for Rapid and Sensitive Diagnosis of Major Viral Pathogens in Calf Diarrhea

Junzhen Chen^1,2^, Yu Wang^1,2^, Rezeguli Aikebaier^1,2^, Haoran Liu^1^, Yingxin Li^1,2^, Li Yang^1,2^, Areayi haiyilati^1,2^, Lixia Wang^1,2^, Qiang Fu^*1,2^ and Huijun Shi^*1,2^

*** Correspondence:** Huijun Shi: [shihuijunmm@163.com](mailto:shihuijunmm@163.com); Qiang Fu: [466183013@qq.com](mailto:466183013@qq.com)

# Figure 1S.


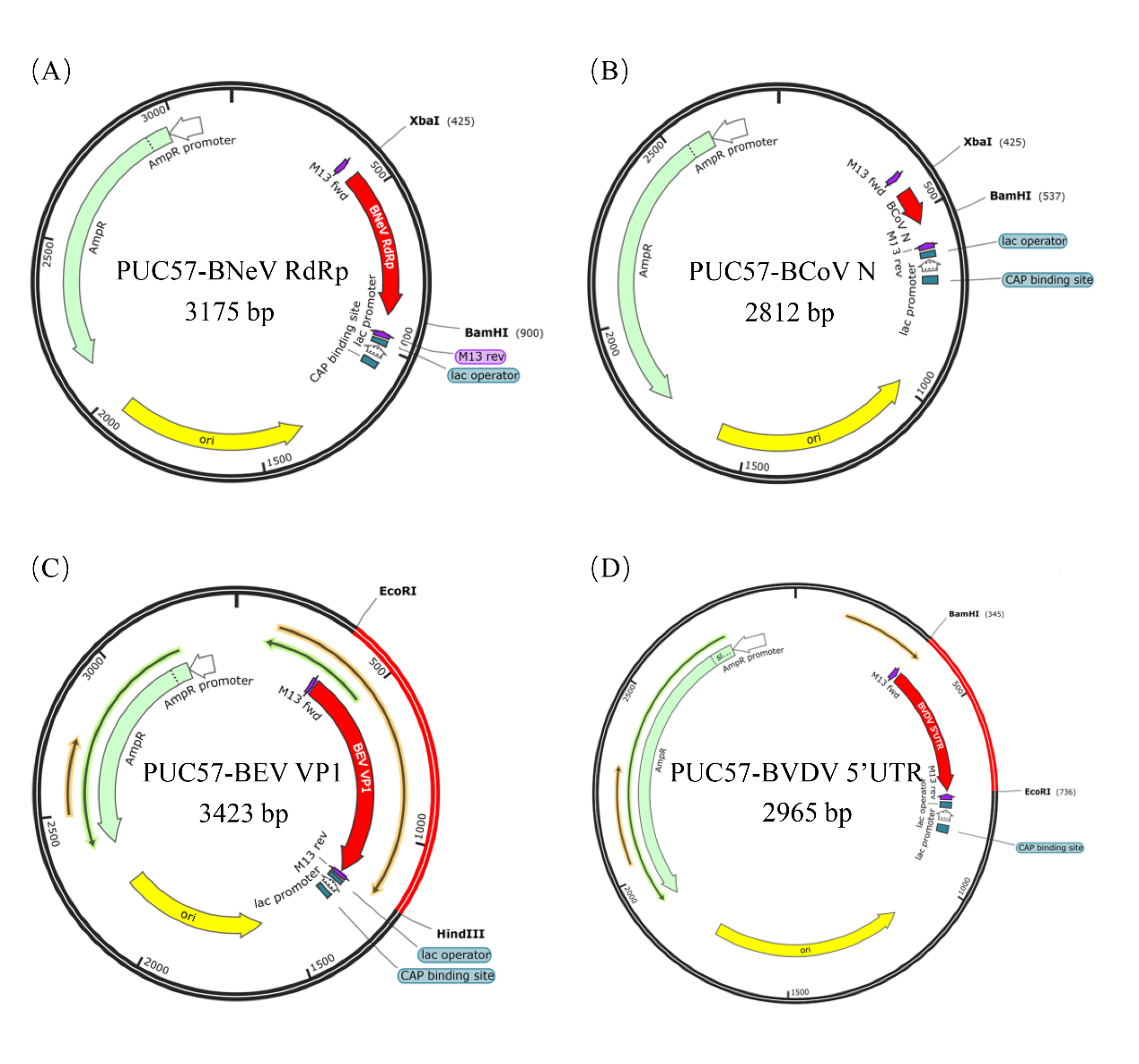


Figure 1S: Plasmid mapping. A: BNeV standard plasmid PUC57-BNeV RdRp mapping; B: BCoV standard plasmid PUC57-BCoV N mapping; C: BEV standard plasmid PUC57-BEV VP1 mapping; D: BVDV standard plasmid PUC57-BVDV 5ʼUTR mapping.

Figure 2S.


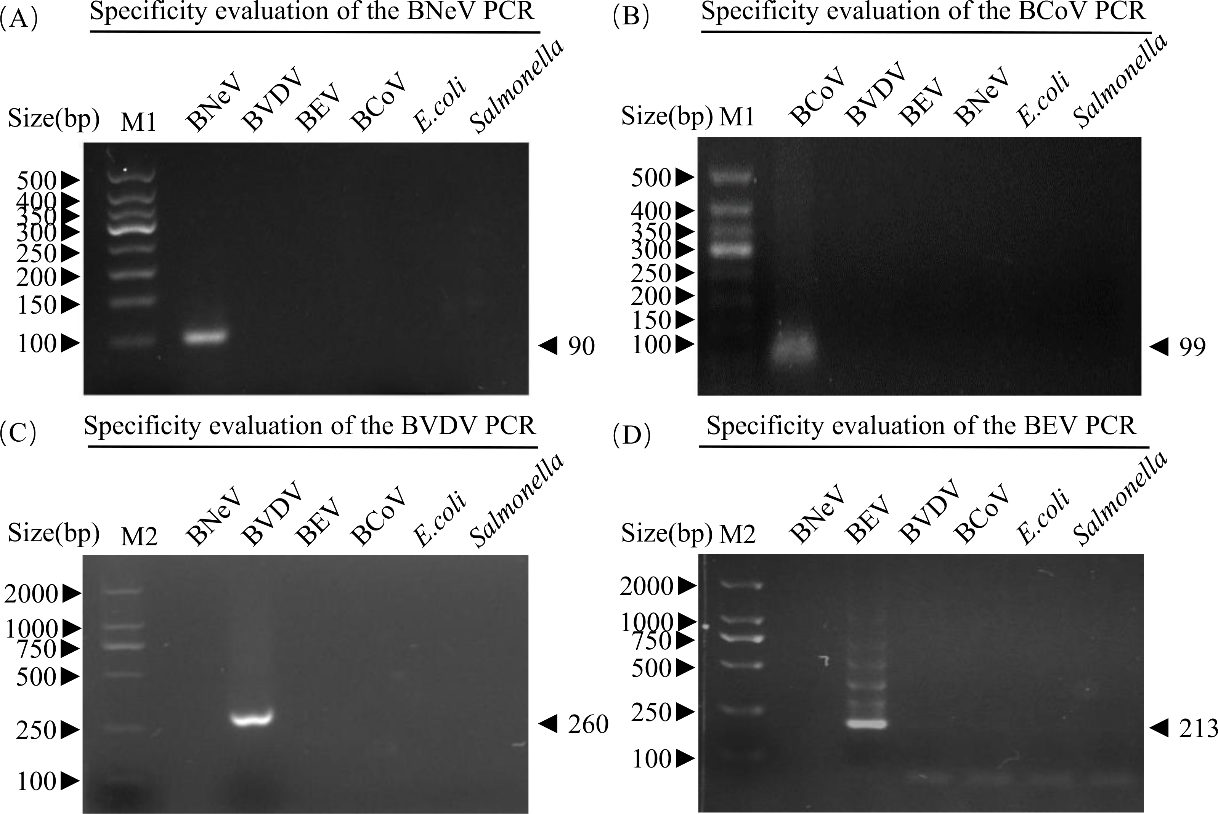


Figure 2S: PCR specificity results. A: BNeV PCR specificity results; B: BCoV PCR specificity results; C: BVDV PCR specificity results; D: BEV PCR assay specificity results. The cDNA of BNeV, BVDV, BEV and BCoV, DNA of *E.coli* and *Salmonella* were used as templates, and ddH_2_O was used as a negative control, respectively, and the results were observed by gel imaging system. M1: DNA marker 50; M2: DL 2000 marke.

# Figure 3S.


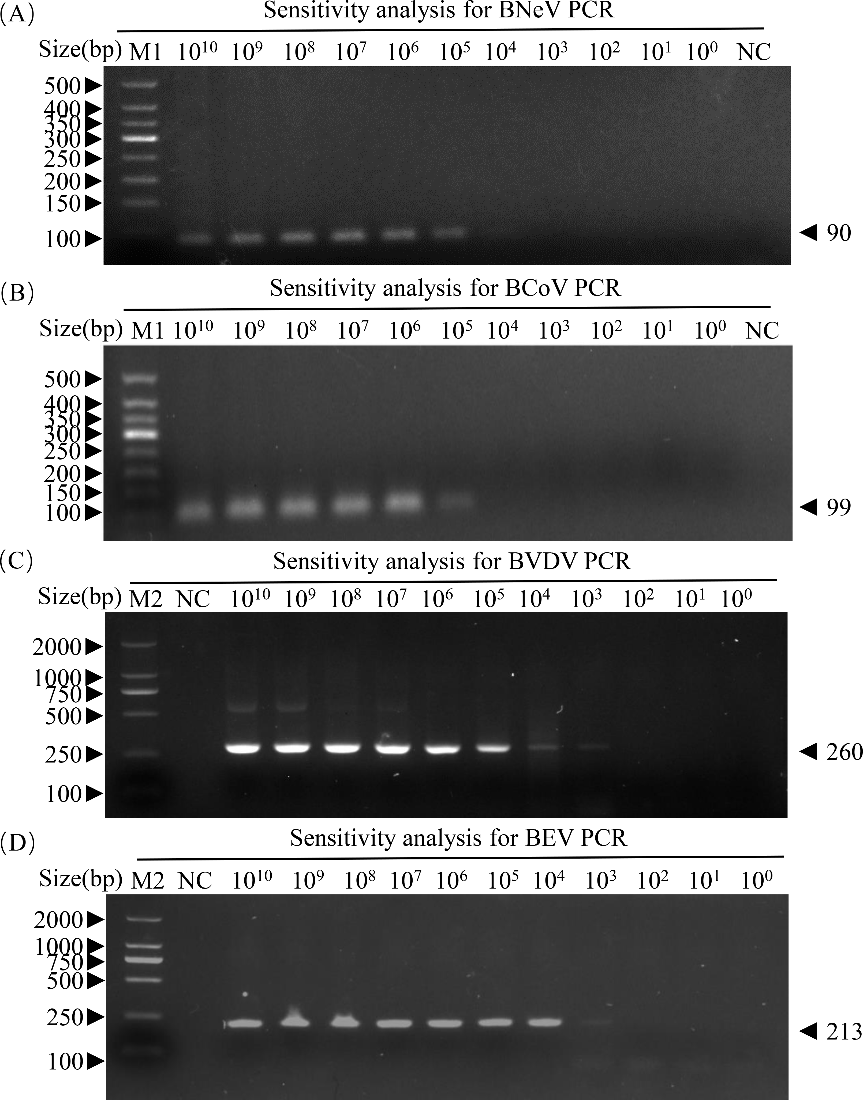


Figure 3S: Sensitivity test results of PCR. A: Sensitivity test results of BNeV PCR; B: Sensitivity test results of BCoV PCR; C: Sensitivity test results of BVDV PCR; D: Sensitivity test results of BEV PCR; Gel imaging of standard plasmids ranging from 1×1010 copies/μL to 1×100 copies/μL respectivelysystem to observe the results. NC: negative control; M1: DNA marker 50; M2: DL 2000 marke.

# Figure 4S.


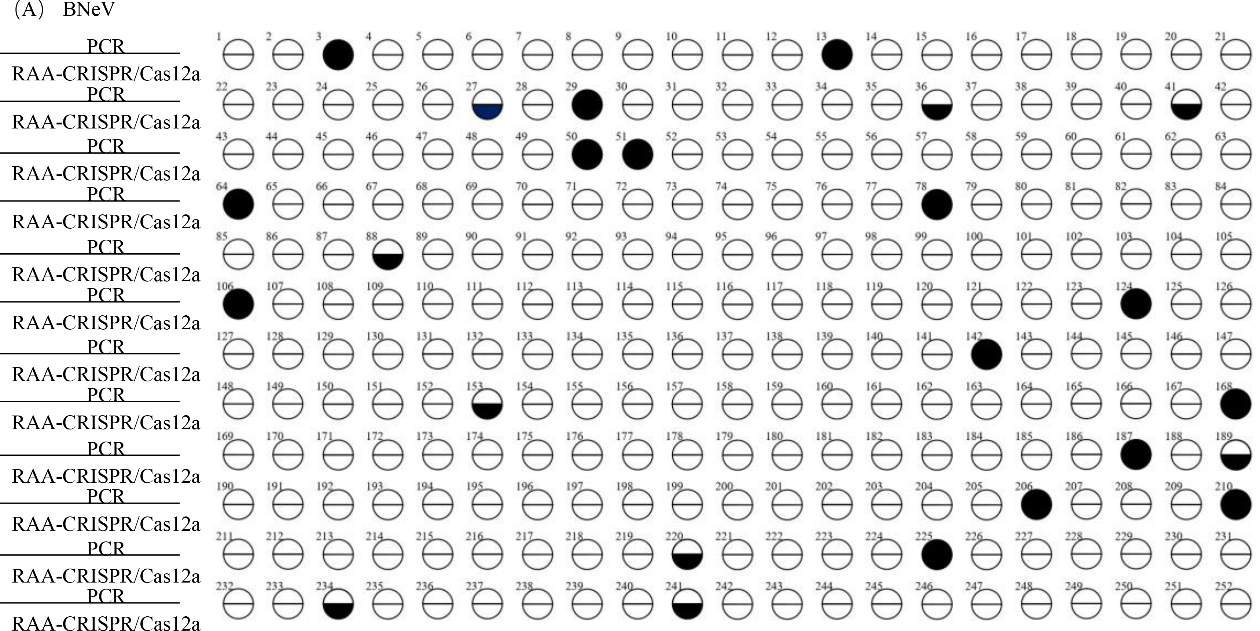


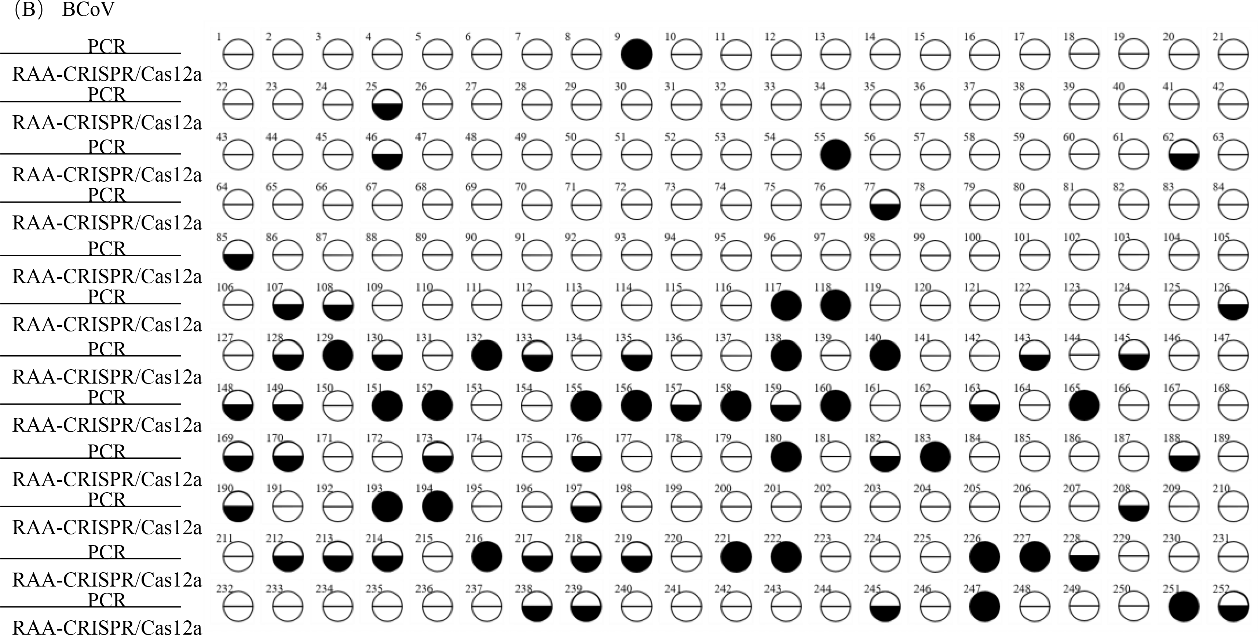


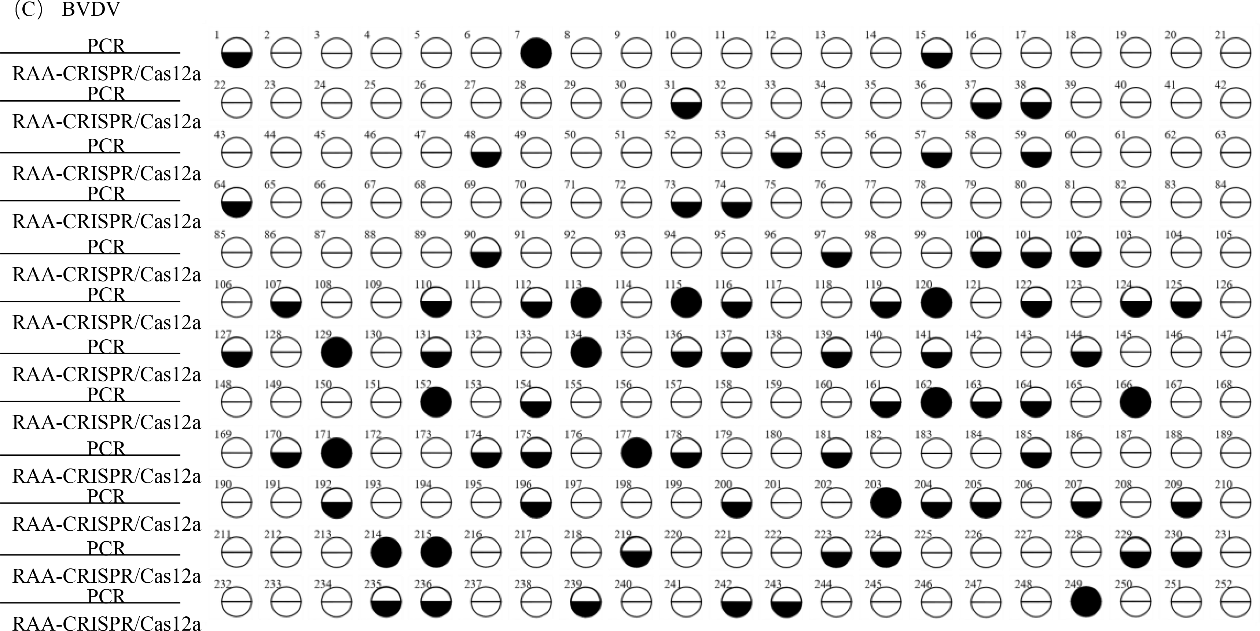


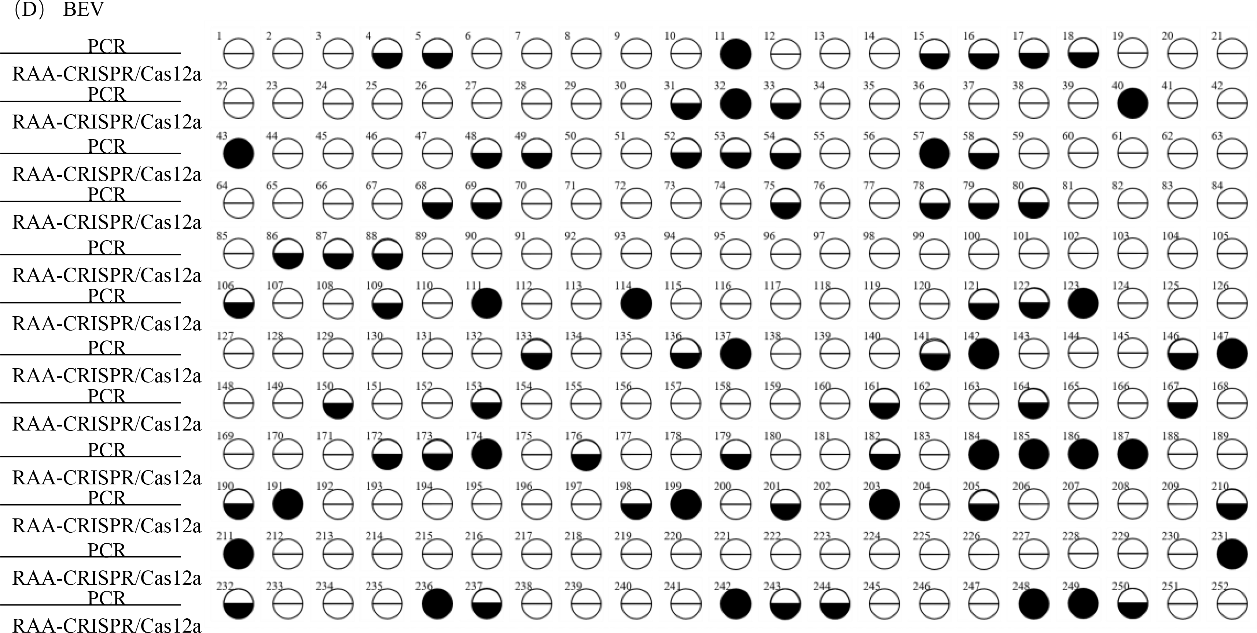


Figure 4S: Clinical detection results. A: BNeV clinical detection results; B: BCoV clinical detection results; C: BVDV; clinical detection results; D: BEV clinical detection results.252 clinical diarrhoea samples (collected from Urumqi, Changji and Bole regions) were tested using established PCR and RAA-CRISPR/Cas12 methods.
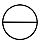
 : PCR and RAA-CRISPR/Cas12 tests were negative;
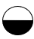
 : PCR showed negative and RAA-CRISPR/Cas12 showed positive;
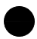
 : PCR and RAA-CRISPR/Cas12 tests were positive.

**
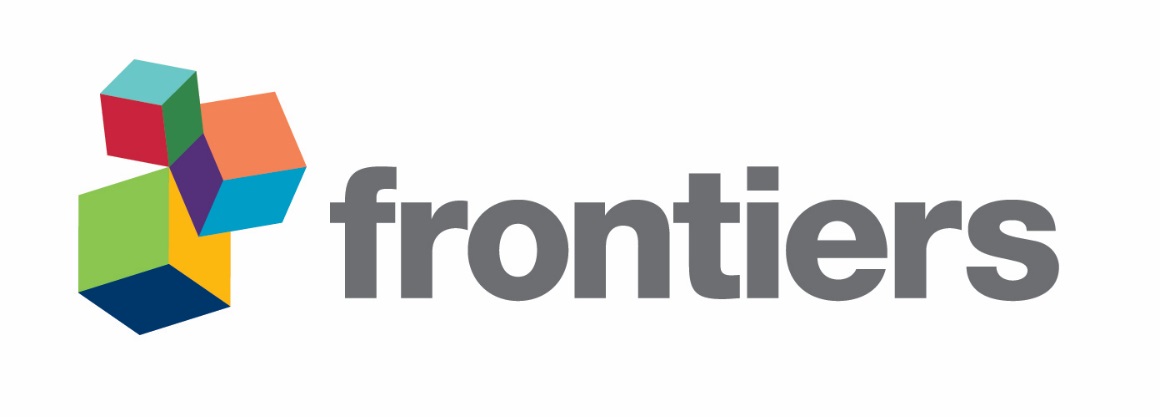
**
